# Motor timing training improves sustained attention performance but not fluid intelligence: near but not far transfer

**DOI:** 10.1007/s00221-020-05780-4

**Published:** 2020-03-23

**Authors:** Olympia Karampela, Guy Madison, Linus Holm

**Affiliations:** Department of Psychology, Umeå University, 901 87 Umeå, Sweden

**Keywords:** Motor timing, Transfer, Near transfer, Intelligence, WAIS, Learning, Tapping, Sensorimotor synchronization, Sustained attention, Cognition

## Abstract

Associations between cognitive and motor timing performance are documented in hundreds of studies. A core finding is a correlation of about − 0.3 to − 0.5 between psychometric intelligence and time interval production variability and reaction time, but the nature of the relationship remains unclear. Here, we investigated whether this relation is subject to near and far transfer across a battery of cognitive and timing tasks. These tasks were administered pre- and post-five daily 30 min sessions of sensorimotor synchronization training with feedback for every interval. The training group exhibited increased sustained attention performance in Conners’ Continuous Performance Test II, but no change in the block design and figure weights subtests from the WAIS-IV. A passive control group exhibited no change in performance on any of the timing or cognitive tests. These findings provide evidence for a direct involvement of sustained attention in motor timing as well as near transfer from synchronization to unpaced serial interval production. Implications for the timing–cognition relationship are discussed in light of various putative timing mechanisms.

## Introduction

Motor timing of brief intervals is a fundamental aspect of everyday activities such as walking, talking, and taking turns in a conversation. Yet, the nature of the mechanisms that achieve adaptive timed behaviour is largely uncharted. A vast number of studies have shown associations of about the same magnitude (*r* = 0.3–0.5) for a range of timed and speeded motor tasks, including reaction time (for recent studies of intelligence and timed behaviours see Der and Deary 2017; Holm et al. 2011; Johnson and Deary 2011; Madison et al. 2009; Ullén et al. 2015; Rammsayer and Troche 2010; Ullén et al. 2008; for reviews see Deary 2000; Doebler and Scheffler 2015; Jensen 2006; Sheppard and Vernon 2008).

The nature of this relationship remains poorly understood, in spite of it being one of the oldest phenomena in academic psychology, first documented by Francis Galton (1883). Empirically, the ability to accurately estimate and produce the duration of a stimulus has been associated with sustained attention and working memory capacity. For example, several timing tasks exhibit an interference effect when non-temporal tasks disrupt timing performance (e.g. Brown 1997; Bååth et al. 2016; Holm et al. 2013; Holm et al. 2017). Therefore, many researchers attribute the interference effect to competition for attentional resources (e.g. Brown 1985; Brown and Benette 2002; Thomas and Weaver 1975; Zakay 1989) which leads to a mutual deterioration of timing performance. This is in line with what attentional models for time perception and estimation would predict, such as the attentional gate model (Zakay and Block 1995). Specifically, this model suggests that when more attention is given to non-temporal information processing, less attentional resources are allocated to temporal processing which results in misperceptions of time.

In the present study, we will employ the sensorimotor synchronization (SMS) and isochronous serial interval production (ISIP) tasks, which form the two phases of the classic synchronization–continuation paradigm. Functional brain imaging studies tend to locate synchronization and continuation tapping processing to brain regions not associated with cognitive processes, such as the basal ganglia, sensorimotor cortex, temporal gyrus, and cerebellum, but are rather inconsistent with regard to other regions (e.g. Chauvigné et al. 2014; Lewis et al. 2004; Lutz et al. 2000; Rao et al. 1997). Structural imaging has however shown that the volume of certain regions in the prefrontal cortex is correlated with both intelligence and ISIP performance (Ullén et al. 2008).

Genetic studies provide another line of evidence for common mechanisms. It is well established that intelligence is strongly heritable (for a summary see Plomin et al. 2016). Twin studies show that also ISIP and reaction time have a heritable component, and that intelligence is genetically correlated with ISIP (Mosing et al. 2016; Ullén et al. 2015) and reaction time (Madison et al. 2016; Sheppard and Vernon 2008).

Yet another source of the associations could be individual differences in specific cognitive abilities, such as sustained attention. Indeed, reaction time (RT) performance has been shown to depend on attention, which is in turn correlated with intelligence (Schweizer et al. 2005; Schweizer and Moosbrugger 2004). Further support for the role of attention on the relationships between intelligence and timing tasks comes from the Worst Performance Rule, which states that performance on the worst trials in reaction timing tasks is most strongly correlated with intelligence (Coyle 2003). The reason could be that those trials reflect attentional lapses, the frequency and magnitude of which is greatest amongst those with lower intelligence.

Evidence from clinical populations also supports the role of attention in time perception and motor timing. It has been shown, for example, that children diagnosed with attentional deficit hyperactivity disorder (ADHD) exhibit problems in both motor timing and cognitive capacities such as attention (Kaplan et al. 1998; Piek et al. 1999). Timing deficits have been consistently reported also in specific neurodegenerative disorders (i.e. Parkinson’s and Huntington disorder), patients with damage in the frontal lobes (Nichelli et al. 2006) as well as depressed patients (Gualtieri et al. 2006). These deficits in the processing of interval durations are attributed to their limited attentional resources or limited working memory capacity—which is well documented in these clinical populations—and leads to more variability in the timing tasks.

Here, we study transfer effects between timing and cognitive functions to further explore these relationships. Transfer occurs when learning in one context enhances (positive transfer) or undermines (negative transfer) a related performance in another context. It is also distinguished between near transfer to closely related contexts and performances, and far transfer to rather different contexts and performances (Perkins and Salomon 1992). Specifically, we examine the extent to which gains from a motor timing training task transfers to a sustained attention task and to fluid intelligence. To this end, we employed a sensorimotor synchronization task for the training sessions, augmented by interval-by-interval auditory and visual feedback. The transfer from the synchronization task to a post-training ISIP task provided a manipulation check for close transfer.

Previous studies have shown that synchronization training improves several aspects of motor and cognitive skills, such as motor coordination and attention (Shaffer et al. 2001; Bartscherer and Dole 2005; Ritter et al. 2013). These studies included children with ADHD and did not include control groups. Here, we therefore extend on previous research by using typically developing adults and employing a passive control group. In addition, we are interested in transfer from training on the synchronization task to the ISIP task. Both these tasks require continuous monitoring of elapsed time, but pose slightly different demands. As follows from the above, synchronization implies feedback-based error correction, whereas ISIP is assumed to constitute an open-loop process, at least at shorter intervals below 700–800 ms (Madison 2006; Madison and Delignières 2009). However, feedback error correction can reduce asynchronies, and have a substantial impact on tempo drift (ibid; Madison 2001; Semjen et al. 2000). Furthermore, participants are required to maintain the target interval in working memory during ISIP, the performance of which should reasonably involve sustained attention. Based on the close similarity of the two tasks, we therefore expect a decrease in ISIP variability post-synchronization training.

The hypotheses were that, compared to the control group, (1) the training group would improve more on the motor timing tasks, (2) the training group would improve more on the sustained attention task, if higher order components such as attention are involved in repetitive motor timing, and that (3) the training group would improve more on the intelligence tasks, if basic neural properties that influence both temporal accuracy and cognitive processes are involved in motor timing. Moreover, we expected (4) to see more reduced ISIP variability in the training group compared to the control group, as 2.5 h of training has been found to decrease variability by about 25% Madison et al. 2013).

## Method

### Participants

Forty students (16 men, 24 women*, M* = 24. 1, SD = 2.45) were recruited via flyers at the Umeå University campus and through an Internet Facebook group. All participants were right-handed and none of them was a professional musician. Inclusion criteria were that the participants were students and between 18 and 35 years old. Exclusion criteria were impaired sight, hearing, and a history of neurological problems. Participants were randomly assigned into a training and a control group. Participants assigned to the training group were paid SEK 500 (~ EUR 50) and those to the control group SEK 300 (~ 30 Euro). The study was approved by the local ethics committee (2012–259-31 Ö).

### Material and tasks

#### Cognitive tasks

##### Block design

In the block design task, participants were required to reproduce a visually presented pattern using red and white coloured blocks. Scores were calculated based on speed and accuracy according to the WAIS–IV manual, yielding a maximum score of 51. The test is primarily a measure of visual–spatial and organizational processing abilities, as well as nonverbal problem-solving skills.

##### Figure weights

In the figure weights, the participant viewed scales with missing weights and selected the weights out of several options to balance a scale. This test is primarily a measurement of quantitative and analogical reasoning. It is also unaffected by language.

##### Sustained attention test

Conners’ Continuous Performance Test II (CPT II) was run as a computerized task on a PC running the Windows 7 operating system. The task consisted of pressing the space bar or clicking the mouse button when any letter except the target letter ‘’X’’ was presented on the screen. The inter-stimulus intervals (ISI) between letters were 1, 2, and 4 s in the same block. There were six blocks with 60 trials each. There were 54 targets consisting of any letter except X (90%) and 6 non-targets (i.e. ‘X’) per block (10%), the order of which was randomly permuted within each block. The CPT II took 14 min to complete. Discriminability (*d*)ʹ was used as a measure of sustained attention performance. Each subject practised the task until the experimenter was confident that the participants had understood the instructions. This test is cited as the most frequently used measure of sustained attention (Riccio et al. 2002). It has been identified as a task sensitive to decrements in performance over time and resistant to training effects.

### Timing tasks

#### Isochronous serial interval production (ISIP)

This task consists of “beating at a regular tempo”, and corresponds to the continuation part of the synchronizing–continuation tapping task (Stevens 1886; Wing and Kristofferson 1973; Madison 2001, 2006). Each trial began with 40 sounds that the participant had to synchronize with to induce the inter-stimulus interval for the ISIP task, and data from the synchronization part were not used. A PC running a real-time operating system (FreeDOS) controlled all aspects of the task. It issued all the sound signals from an Alesis DM5 sound module, connected via a MIDI interface. The sound with which to synchronize was Prc/Claves, presented through Peltor HTB7A headphones at 84 dBA SPL.

Each participant was tested individually, sitting upright on a chair with the feet on the floor. A computer screen was positioned at eye height and slightly to the left of the participant. An electronic drum pad was placed to the participant’s right front side at a comfortable position, at which to beat a drumstick with the right hand. After the participant had synchronized with 40 sounds, the sounds ceased, and the participant continued to produce 200 responses without any interruption and with the same interval, until a stop signal sounded. There were two such trials for each of the four prescribed inter-response intervals (IRI): 524, 733, 1024, and 1431 ms. If the mean IRI across the last six responses was shorter than 66% of the ISI or if more than three times the ISI had elapsed since the last response, the computer generated a warning signal. This signal consisted of a bongo drum presented with a sound pressure of 78 dB SPL, repeated with random intervals in the range of 40–100 ms, which was thought to constitute minimal temporal information and thus distraction of the timing behaviour. Together with the instructions, the eight trials took about 35 min to complete.

#### Sensorimotor synchronization training

This task setup involved the same headphones and drum pad as was used in the ISIP task. The two setups differed in that the software was run on a PC with the Windows 7 operating system and an LCD screen with a resolution of 1920 × 1080 pixels was used for visual feedback. Stimulus sounds were produced by an Arduino Uno 1.6.3 microcomputer and consisted of a 262 Hz sine tone lasting 50 ms, presented at 1024 ms ISI. Participants used a drumstick and their beats were recorded and time-stamped by the Arduino in real time and sent to the Windows PC for processing in an in-house developed Matlab code running in the Matlab 2014b environment. The participant feedback based on the beat records (explained below) was supplied via the Matlab code on the PC.

The participants produced 200 synchronization intervals per trial. There were five trials per session and short breaks between each trial. Participants received visual and auditory feedback on each interval production. Computation of asynchrony and presentation of feedback was done via in-house developed Matlab code, using the PsychToolbox-3 methods library (Brainard 1997; Pelli 1997).

**Fig. 1:**
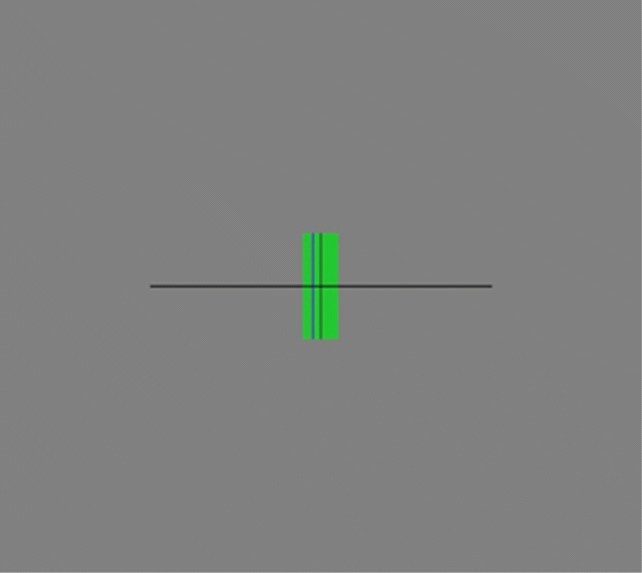
Display of the visual feedback when the response was within 5% of the metronome beep

Throughout the synchronization task, the screen displayed a horizontal line intersected by a vertical line at the central meridian of the screen, as illustrated in Fig. 1. The visual feedback was presented as a vertical bar moving proportionally with the participant’s asynchrony along the meridian: to the left if ahead of the metronome, and to the right if behind in time. If participants were within 5% of the metronome beep, a green box was displayed around the vertical bar.

**Fig. 2:**
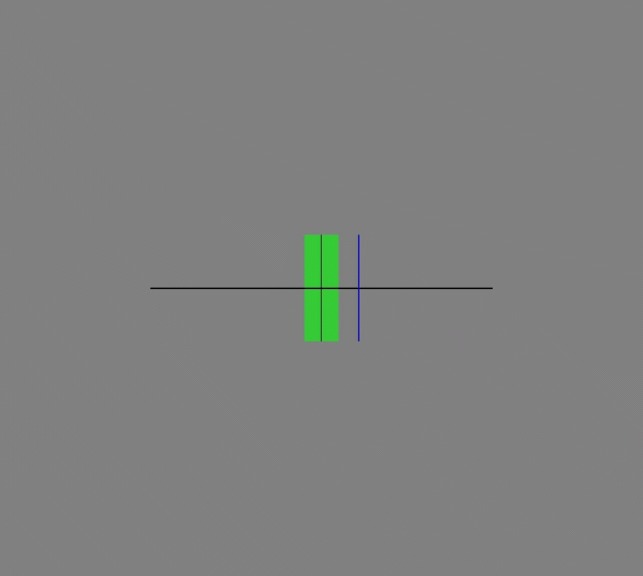
Display of the visual feedback when the response was more than 5% after the metronome beep

The auditory feedback was presented via loudspeakers in front of the participant and consisted of different frequencies for the feedback sound. This sound was a sine tone with a duration of 100 ms, which started simultaneously with the response. The frequency of the feedback sound was 523 Hz when the response was within 51 ms of the stimulus, corresponding to 5 percent of the ISI, 740 Hz if the response was too early (between 51 and 512 ms before the stimulus sound) and 370 Hz if it was too late (between 51 and 512 ms after the metronome) (Fig. 2). Each training session lasted around 30 min. At the end of each session, participants were shown their results depicted in a graph on the computer screen.

#### Dependent measures computation

Performance in the synchronization training and ISIP tasks was defined as the standard deviation of the mean inter-response interval. The ISIP variability was also gauged by the so-called local variability, which eliminates the influence of the gradual drift in IRI that occurs when intervals are produced without any external guiding signal (Madison 2006). It is computed according to Eq. 1:

1 $${\text{Local}} = \sqrt {\frac{{\mathop \sum \nolimits_{1}^{N - 2} \left( {x_{i + 2} - x_{i} } \right)^{2} }}{{2\left( {N - 2} \right)}}} ,$$

where *x*_i_ is the duration of the temporal interval between response *i* and response *i* + 1, $\overline{x}$ is the mean of all intervals of the trial and *N* is the number of intervals in a trial. In other words, the expression inside the square root is a variance measure based on lag 2 local differences between data points.

Discriminability *d*ʹ is a measure of the difference between the signal plus noise (non-X) and noise (X) distributions. As such, *d*ʹ provides a means for assessing an individual's discriminative sensitivity, since, in general, the greater the difference between the signal plus noise and noise distributions, the better is the ability to distinguish between the stimuli. We computed *d*ʹ as *Z*(hit rate) − *Z*(false alarm rate), where a hit refers to responding in the presence of a non-X and a false alarm refers to responding in the presence of an X. Under the assumption of normality, then, the difference between the *z* scores indicates the distance between the means of signal + noise and noise distributions.

### Procedure

Participants in both the training and control group were informed that they would partake in a series of cognitive and motor tasks, and gave written informed consent prior to the study. All participants were tested in pre- and post-test sessions, each with four tasks that occurred in the following order: block design and figure weights subtests from the Wechsler Adult Intelligent Scale (WAIS IV) battery, Conner’s Continuous Performance Test II, and the ISIP task. The order of the tasks was the same for all participants both in the pre- and post-sessions, to minimize variability. Between the pre- and post-tests, the training group trained synchronization for a total of 150 min, in 30 min sessions on five separate days.

## Results

One participant was excluded from further analysis because her synchronization variability was more than two standard deviations above the mean of the rest of the training group.

### Training

**Fig. 3:**
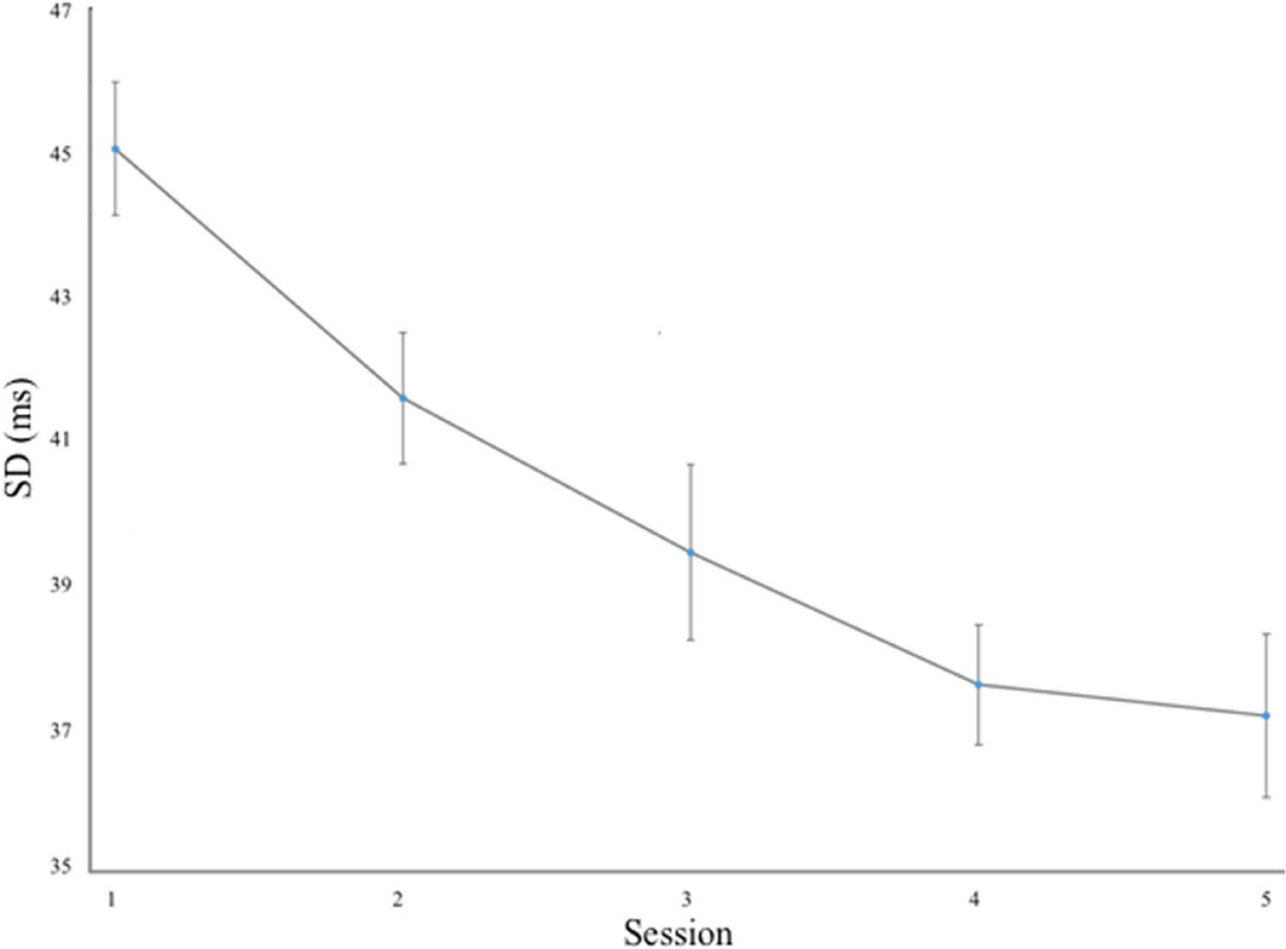
Synchronization variability as a function of training. Error bars indicate one standard error of the mean (SEM)

The variability across the remaining participants decreased monotonically from 45.0 ms SD at the first session to 37.1 ms in the last session, as depicted in Fig. 3. A dependent *t* test indicated a significant difference between the first and last training session, *t*(18) = 6.27, *p* < 0.05.

### Cognitive tasks

Table 1 shows that the training and the control group performed at a similar level in all the cognitive tasks except sustained attention, where the training group had improved post-training. Effect size measures were computed using partial eta squared (*η*_p_^2^). For block design, a mixed 2 (group) × 2 (pre–post) ANOVA indicated a main effect of pre–post, *F*_1, 38_ = 0.59.672, *p* < 0.05, *η*_p_^2^ = 0.611, but not of group, *F*_1, 38_ = 0.203, *p* = 0.655, *η*_p_^2^ = 0.005, or of the session × group interaction, *F*_1, 38_ = 0.1.247, *p* = 0.271, *η*_p_^2^ = 0.032, suggesting that both groups improved about equally across sessions. Similarly, figure weights test ANOVA exhibited a main effect of pre–post *F*_1, 38_ = 24.91 *p* < 0.05 *η*_p_^2^ = 0.396, but not of group, *F*_1, 38_ = 0.2.682, *p* = 0.110, *η*_p_^2^ = 0.066, or of their interaction, *F*_1, 38_ = 3.177, *p* = 0.083, *η*_p_^2^ = 0.077.

**Table 1:** Descriptive statistics for the variables block design, figure weights and sustained attention (*d*ʹ prime), for the training and the control group in the pre- and post-tests, respectively

	Block design			Figure weights		Sustained attention	
	Pre	Post		Pre	Post	Pre	Post
Training							
Mean	38.1	43		20.8	23.7	0.68	1.05
SD	6.05	5.4		4.1	2.20	0.42	0.45
Control							
Mean	39.5		43.1	19.9	21.3	0.75	0.85
SD	7.2		5.8	3.9	3.14	0.35	0.32

**Fig. 4:**
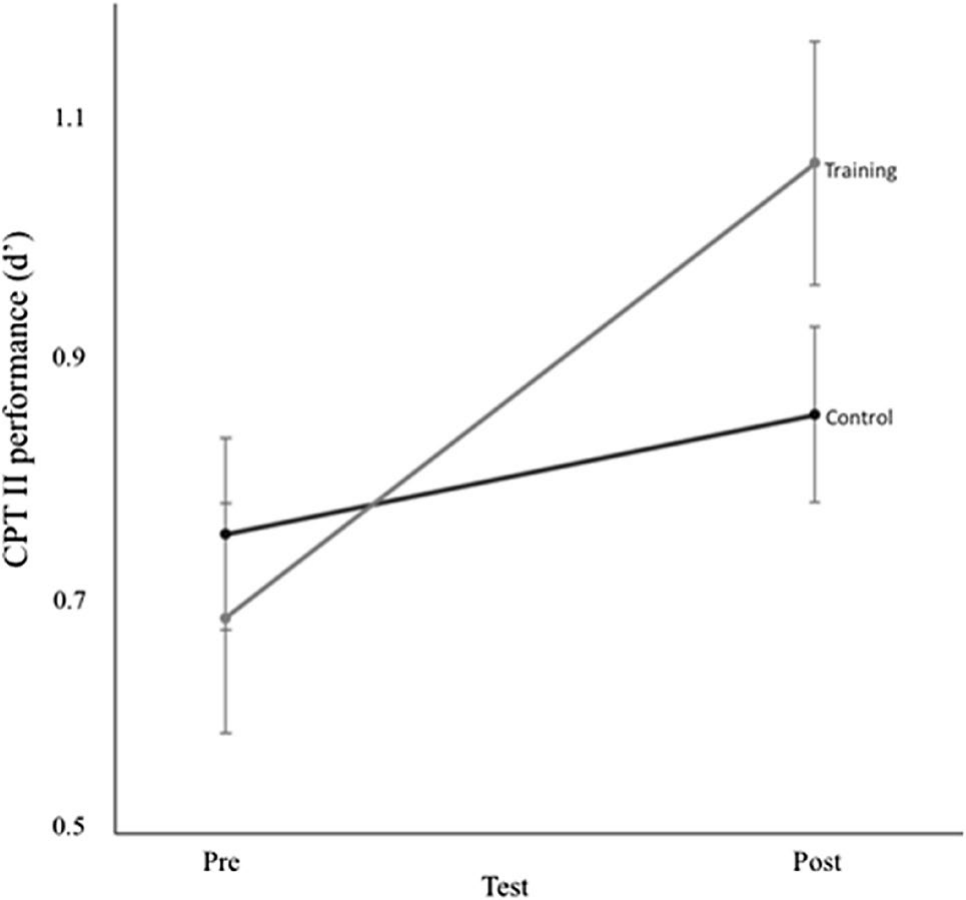
Performance on sustained attention (CPT II), as a function of test session for the control and training group. Error bars express one SEM

For the sustained attention task, there was a significant main effect of session, *F*_1, 38_ = 24.910, *p* < 0.05, *η*_p_^2^ = 0.396, but not of group *F*_1, 38_ = 2.682, *p* = 0.110, *η*_p_^2^ = 0.066. Critically, there was a significant session × group interaction, *F*_1, 38_ = 5.865, *p* = 0.0.02, *η*_p_^2^ = 0.134, indicating that the training group improved more than the control group, as depicted in Fig. 4.

To test whether the sustained attention performance improvement could be predicted from individual synchronization training effects, we computed Pearson’s correlation between the difference in SD between the first and last training sessions, and the *d*ʹ difference in the sustained attention task between the pre- and post-test. This correlation was positive but non-significant (*r* = 0.109, *p* > 0.05).

The response sequences were quite long (200 intervals), which allows for drift that might inflate the standard deviation, as mentioned above. We therefore focus on the local estimates (Eq. 1), but include the raw SDs for transparency in Table 2, which shows SD and local separately for the control and training group and the pre- and post-sessions. A mixed-model 2 (group) × 2 (pre–post) × 4 (ISI) factorial ANOVA with Local as dependent variable indicated a significant main effect of ISI, *F*_3, 114_ = 245.1, *p* < 0.00001, but not of group, *F*_1, 38_ = 2.7, *p* = 0.011, or pre–post, *F*_1, 38_ = 4.06, *p* = 0.0.051, which was just short of significance. Critically, the pre–post × group interaction was statistically significant, *F*_1, 38_ = 5.92, *p* = 0.02, showing that the training but not the control group decreased their variability for all IOI, as indicated by *F* values below 1 for all other interactions. The massive effect of ISI is likely to distort the much weaker interactions because of the scalar timing effect (Gibbon et al. 1997; Madison 2004b). The coefficient of variation (CV) is therefore a more appropriate metric, that is, Local divided by the IRI for each trial sequence, multiplied by 100 to be expressed in percent. An identical ANOVA with CV Local as dependent variable indicated significant main effects of ISI, *F*_3, 114_ = 6.92*, p* = 0.00025, and pre–post, 7.66 *p* = 0.009, but not of group, *F*_1, 38_ = 1.99, *p* = 0.17. Again, the critical pre–post × group interaction was significant, *F*_1, 38_ = 11.3, *p* = 0.0018, *η*_p_^2^ = 0.23. All of the remaining interactions exhibited *F* values below 1.5, *p* > 0.25. The data suggest that the difference between 1024 and 1431 ms violate the scalar principle, consistent with previous studies (Madison 2001, 2004b, 2006; Grondin et al. 2015). We tested this specifically for CV Local by comparing adjacent levels of ISI for each group and pre–post session separately, which amounts to 12 contrasts in total. Post hoc tests showed no significant effects for the control group. However, the training group exhibited a significant difference between 1024 and 1431 ms, but not for any other ISI levels, both pre-training, *F*_1, 38_ = 7.48, *p* = 0.0094, and post-training, *F*_1, 38_ = 11.82, *p* = 0.0014.

**Table 2:** Timing variability for the ISIP task for the control and the training group in pre- and post-tests

	Inter-stimulus interval (ISI)											
	524 ms			733 ms			1024 ms			1431 ms		
	SD	Local	CV	SD	Local	CV	SD	Local	CV	SD	Local	CV
Control												
Pre	28.1	23.7	4.49	41.8	33.5	4.52	67	49.9	4.71	111.1	74.6	4.91
Post	31.1	24.2	4.47	44.7	35.6	4.72	68.5	50.7	4.75	106.6	73.9	5.06
Training												
Pre	30.0	23.8	4.55	45.9	34.56	4.74	69.6	46.5	4.57	111.2	73.6	5.32
Post	29.1	20.8	3.90	36.6	26.80	3.56	62.2	38.4	3.53	94.2	64.5	4.41

*SD* standard deviation. Local variability excludes drift (Eq. 1), and is otherwise comparable to SD. *CV* coefficient of variation for local, i.e. local/IRI × 100

## Discussion

The aim of this study was to test the involvement of cognitive capacities in motor timing by assessing transfer effects from motor timing training to sustained attention as well as the transfer from a sensorimotor synchronization training to ISIP. The hypothesis that the timing trained group would improve more in the sustained attention task compared to the control group was borne out by the results. Furthermore, we found no evidence for timing training improvements on measures of other types of cognitive ability employed in the study. The small improvements in block design task and figures weight task are likely trivial test-taking effects and do not differ across the training and control group. Finally, we found an improvement in the ISIP task following sensorimotor synchronization training, indicating near transfer to a timing task conceivably involving slightly higher working memory demand than synchronization tapping.

Studies on working memory-training effects offer a rather inconsistent picture. A meta-analysis (Schwaighofer et al. 2015) showed that training of working memory yielded both immediate and sustained near-transfer effects to both short-term memory and working memory components. Other studies have reported far transfer to untrained abilities, such as reasoning) (Jaeggi et al. 2008), as well as both near and far-transfer effects as a result of training skills related to working memory (WM) (Brehmer et al. 2012). Yet other training studies report no transfer at all (Redick et al. 2013; Thompson et al. 2013). Finally, studies with older adults have typically reported smaller or null transfer effects, as compared to younger adults (Brehmer et al. 2012; Buschkühl et al. 2008; Dahlin et al. 2008a; Li et al. 2008).

With regard to motor timing and sustained attention transfer, this effect might be due to the close match in response requirements between the synchronization training and the sustained attention task. Specifically, timed behaviour appears to for the most part involve active regulation in terms of error correction with respect to previous intervals or asynchronies, and to a memorized model of the target interval (Staddon 2005). This would involve attentive processes over time, at least for intervals longer than about 500 ms (Madison 2004a, 2006; Madison and Delignières 2009). The CPT II also involves regulation of behaviour over time. This interpretation is in line with Dahlin et al. (2008b), who argued that transfer can occur if the criterion and transfer tasks engage specific overlapping processing components and brain regions. Thus, the motor timing training improvement might reflect improved ability to maintain focus and allocate sustained attention more efficiently in the service of regulating timed behaviour. In other words, we might have trained sustained attention and not motor timing ability per se through the synchronization training. Therefore, it seems reasonable to view our results as near transfer rather than far transfer.

Overall, this study provides support that repetitive motor timing in the range from a few hundred milliseconds to a few seconds range employs regulation subject to controlled cognitive processes, specifically to sustained attention. Another relevant line of research suggests that the speed and accuracy of visual and visuo-motor processing is predicted by efficient modulation of attentional resources (Klimesch et al. 1998; Serences and Yantis 2006; van Dijk et al. 2008). This indicates that the nervous system’s ability to modulate its representation of time is highly influenced by the attentional state of the observer. Accordingly, Krampe et al. (2005) proposed that the timing and sequencing of paced movement is produced via two distinct processes: a low-level timing process and a higher-level timing process operating within the larger system of executive control. During the production of self-paced interval sequences, executive functions control the low-level timing mechanism by, for example, updating and maintaining the temporal stimuli and supervising changes in the movement production.

With regard to the transfer from the synchronization training task to the ISIP task, one explanation could be that the feedback-based error correction led to a better calibrated internal model of error correction. Such an improved model should then affect the local interval-to-interval ISIP accuracy. In other words, training reduced the motor implementation error expressed as reduced timing variability both in the synchronization and ISIP tasks. Another explanation could be that training in the sensorimotor synchronization task increased predictive control. Given the fact that the sensorimotor synchronization task is a form of referential behaviour (Pressing 1999) in which an action is coordinated with an external predictable event, it seems possible that predictive control had been increased during the sensorimotor synchronization, affecting also the internally generated intervals during the ISIP.

Based on our previous argument that cognitive control expressed via sustained attention seems to be involved in the sensorimotor synchronization timing task, it might be possible that the same relationship holds for the ISIP task. It seems likely that an ISIP task involves cognitive control, because regular motor output must be maintained and successfully represented in working memory in the absence of any external cues (Jones et al. 2011). In concert with the argumentation above, Witt and Stevens (2013) provided direct evidence for the contribution of top-down control during different phases of a unimanual, auditory-paced synchronization task. By investigating changes in dynamic causal modelling (DCM) they measured top-down control of sensorimotor timing between putatively higher-level cognitive (e.g. prefrontal) and lower-level sensory and motor areas. They found that subjects who performed better in keeping the interval constant in the absence of the auditory cue relied more on top-down control of the motor and sensory regions, while those with less accurate performance relied more on sensory driven, bottom-up control of the motor cortex (Witt and Stevens 2013).

Finally, the present results bear on the established relationship between timing and intelligence (Madison et al. 2009), suggesting that top-down influences might also contribute to correlations between intelligence and motor timing. Such influence could operate in several different ways. For example, more intelligent people could perform better in timed tasks due to better cognitive control mechanisms that are used both in timing tasks and in problem solving. For example, they might perform better in timing tasks because they have better focus and not so many lapses of attention (Ullén et al. 2008). That is also in line with several studies which have shown an association between intelligence and sustained attention (Buehner et al. 2006; Ren et al. 2013; Schweizer 2000) as well as with studies which propose that that fluctuations or lapses in sustained attention are related to executive control and fluid intelligence (Unsworth et al. 2010).

While the present study strongly implicates sustained attention in regulating motor timing, the precise operation of that regulation is still unclear. One avenue of future investigation would therefore be to make closer analyses and modelling of motor timing under divided attention or through the course of motor timing training.
